# Changes in the Transcriptome Profile in Young Chickens after Infection with LaSota Newcastle Disease Virus

**DOI:** 10.3390/vaccines12060592

**Published:** 2024-05-30

**Authors:** Taina S. B. Lopes, Jannis Nankemann, Cassandra Breedlove, Andrea Pietruska, Raimundo Espejo, Camila Cuadrado, Ruediger Hauck

**Affiliations:** 1 Department of Pathobiology, College of Veterinary Medicine, Auburn University, Auburn, AL 36849, USA; tsl0026@auburn.edu (T.S.B.L.);; 2 Department of Poultry Science, College of Agriculture, Auburn University, Auburn, AL 36849, USA

**Keywords:** vaccine, immunogenomics, gene regulation, pathway analysis, bioinformatics

## Abstract

Understanding gene expression changes in chicks after vaccination against Newcastle Disease (ND) can reveal vaccine biomarkers. There are limited data on chicks’ early immune response after ND vaccination. Two trials focused on this knowledge gap. In experiment one, 42 13-day-old specific-pathogen-free (SPF) chicks were used. Harderian glands (Hgs) and tracheas (Tcs) from five birds per group were sampled at 12, 24, and 48 h post-vaccination (hpv) to evaluate the gene transcription levels by RNA sequencing (RNA-seq) and RT-qPCR. The results of RNA-seq were compared by glmFTest, while results of RT-qPCR were compared by t-test. With RNA-seq, a significant up-regulation of interferon-related genes along with JAK-STAT signaling pathway regulation was observed in the Hgs at 24 hpv. None of the differentially expressed genes (DEGs) identified by RNA-seq were positive for RT-qPCR. Experiment 2 used 112 SPF and commercial chickens that were 1 day old and 14 days old. Only the commercial birds had maternal antibodies for Newcastle Disease virus (NDV). By RNA-seq, 20 core DEGs associated with innate immunity and viral genome replication inhibition were identified. Genes previously unlinked to NDV response, such as USP41, were identified. This research present genes with potential as immunity biomarkers for vaccines, yet further investigation is needed to correlate the core gene expression with viral shedding post-vaccination.

## 1. Introduction

Newcastle disease (ND) is a respiratory or systemic disease caused by avian orthoavulavirus serotype 1, commonly referred to as Newcastle Disease virus (NDV) [1]. ND has caused financial losses due to increases in bird mortality in poultry flocks, expenses related to outbreak eradication, and trade restrictions during outbreaks [2,3]. Current ND vaccines do not prevent NDV infection and shedding and thus might mask outbreaks [4]. Improved vaccines would be helpful to avoid production and economic losses. Understanding the avian immune response after infection or vaccination with NDV live vaccines is necessary for the rational design and testing of vaccines.

Understanding early gene expression changes in chickens after vaccination against ND can identify immune-related genes linked to clinical disease prevention and virus shedding that could be used as immunity biomarkers. Transcriptome analysis allows for in-depth analysis of gene expression [5] and permits identifying changes in the expression of immune-related genes after viral exposure with high sensitivity [6], without the need to pre-select certain genes and thus bias the results. Previous studies have identified differentially expressed genes in the Bursa, Harderian glands (Hgs), lungs, spleen and thymus at 2, 3, 6, and 10 days post-infection (dpi) in birds at 21 to 42 days of age [7,8,9,10,11,12,13]. However, there is little information about even earlier innate immune response in the first few hours after vaccination. To understand the early innate immune response, it is necessary to identify transcriptomic changes in tissues that are considered NDV entry sites. In this sense, lymphoid-associated tissues and upper respiratory tract elements such as Hgs and tracheas (Tcs) have a key role.

Knowing the early innate immune response in day-old chicks after ND vaccination is also important because, in the field, birds are frequently vaccinated right after hatching. Considering that the immune system of chickens is only fully mature after four to seven weeks of age [14], results obtained in older birds might not be applicable for hatchling chicks. Furthermore, due to the widespread use of vaccines, chicks are likely to have maternal antibodies against NDV, and there is no consensus if its presence can alter the immune response to ND vaccine [15,16].

In the current study, two experiments investigated the early innate immune response by transcriptome analysis in chicks upon vaccination with the NDV LaSota strain. In experiment 1, 13-day-old specific-pathogen-free (SPF) chickens were vaccinated. Changes in gene transcription 12, 24 and 48 h post-vaccination (hpv) were investigated by transcriptome analysis using RNA sequencing (RNA-seq) and RT-qPCR. The innate immune genes identified by both methods were compared. Experiment 2 studied the transcriptome 24 and 48 hpv of 1-day-old and 14-day-old chickens with or without maternal antibodies against NDV. The main objective was to identify target innate immune genes expressed shortly after vaccination and recognize potential candidates to be further tested as immunity biomarkers for ND vaccines.

## 2. Materials and Methods

Birds were kept in Horsfall-type isolators in biosafety level 2 facilities. All birds in both experiments were fed a standard commercial chicken feed that met the National Research Council’s recommended minimum nutrient requirements for laying hens [17]. Feed and water were provided ad libitum. The housing temperature and humidity were set following the breed rearing manual. Birds were randomly selected from each isolator and euthanized with carbon dioxide for sample collection.

**Experimental design of experiment 1.** A total of 42 white leghorn specific-pathogen-free (SPF) fertile eggs (AVS Bio, Catskill, NY, USA) were hatched and randomly distributed in 2 groups of 21 birds each. At 13 days of age, each bird of one group received 10^7^ of a 50 percent embryo infectious dose (EID_50_) of a commercial NDV LaSota vaccine of 100 µL via the ocular route (50 µL in each eye). The other group served as an unvaccinated control and was mock-vaccinated with phosphate-buffered saline solution. Moreover, 12, 24, and 48 hpv, five birds per group were euthanized for sampling collection. Both Hgs and the whole Tc from the same bird were collected, wrapped in individual aluminum foils, and flash-frozen on dry ice immediately after collection. Samples were then stored at −80 °C until further processing.

**RNA extraction and RNA-seq.** Total RNA was extracted from Hgs using the RNeasy kit (Qiagen, Hilden, Germany) in a QIAcube automated extraction system (Qiagen) accordingly to the manufacturer’s protocol. For the Tcs, total RNA was extracted using the Quick RNA Miniprep kit (Zymo Research, Irvine, CA, USA) following the manufacturer’s instructions.

The RNA concentration of each sample was measured by spectrophotometry (Nanodrop 2000, Thermo Scientific, Waltham, MA, USA). Three replicates with the highest RNA concentration and A_260_:A_280_ and A_260_:A_230_ ratios in the 2.0 to 2.2 range were selected. mRNA library preparation was performed by poly A enrichment and sequencing on a NovaSeq PE150 system (Novogene Corporation Inc., Sacramento, CA, USA). The sequencing data were uploaded to the Sequence Read Archive database (SRA accession number SAMN40643659).

**Differential expression of genes, gene ontology, Kyoto Encyclopedia of Genes pathway enrichment, and protein–protein interaction analysis.** The index for the chicken reference genome (NCBI GenBank accession number GCF_016699485.2) was generated using HISAT2 v2.0.5 [18] with default parameters. Raw reads were quality trimmed using Trimmomatic v0.39 [19] with standard settings prior to downstream analysis. Trimmed reads were aligned to the reference genome using HISAT2, and the read counts for gene expression were obtained using HTSeq v1.13 [20]. Counted mapped reads were further analyzed using the edgeR package v4.0 [21] in R v4.3.1 [22]. The reads were filtered to exclude genes with low counts and normalized by the trimmed mean of M-values method (TMM) [23]. For each organ and timepoint, the expression levels from the vaccinated groups were compared to unvaccinated groups, considering three replicates per group, by Fishers’ exact test. The differential gene expression analysis results were considered significant with an adjusted *p*-value < 0.05 for the false discovery rate (FDR) and logarithm of fold change (logFC) > ±0.5.

The top-50 differentially expressed genes (DEGs) from each comparison were selected based on their *p*-value. They were used as input for mapping gene ontology (GO) pathways related to each gene based on the genome-wide annotation from the chicken package (org.Gg.eg.db) by Bioconductor v3.18 [24]. For GO and Kyoto Encyclopedia of Genes (KEGG) pathway enrichment analysis, limma v4.3 [25] was used to identify up- and down-regulated pathways. The GO terms and KEGG pathways with *p*-values < 0.05 were considered significant. In case the output result showed more than 50 significantly regulated pathways, only the top 50, based on their *p*-value, were considered.

In addition, the top-ranked DEGs were used as input into the STRING database [26] for protein–protein analysis (PPI). The “multiple proteins” function was used with *Gallus gallus* as the target organism. The program standard settings were used to determine the PPI network, with a minimum required interaction score of 0.400, excluding non-experimentally determined protein interactions. The PPI network for each significant contrast was imported to CytoScape, v3.10.1 [27]. The String Enrichment plugin and the String: protein query database from STRINGapp v2.0.1 [28] were used to retrieve the functionality enrichment map. The genome was used as the background. The connectivity cutoff (Jaccard similarity) was 0.8, and the node cutoff *p*-value was < 0.05. CytoCluster app v2.1.0 [29] with the ClusterONE algorithm [30] was used to identify protein clusters. Minimum size = 5, minimum density = 0.05, edge weights = combined_score, EnrichmenMap: similarity_coefficient, similarity = Jaccard similarity, and overlap threshold = 0.8 were the settings used to identify clusters linked to immune-related pathways.

**Differential expression of genes associated with immune response by RT-qPCR.** cDNA was synthesized from three samples per group, and total RNA was obtained using random hexamers and oligo-DT primers with the RT^2^ First Strand Kit (Qiagen) following the manufacturer’s instructions. The quantity and quality of the resulting cDNA were measured by spectrophotometry. All samples were normalized to a concentration of 0.5 µg of total RNA followed by dilution to a total volume of 111 µL with RNAse-free water and 2× RT^2^ SYBR green master mix and stored at −20 °C until further analysis. For the RT-qPCR, the RT^2^ Profiler™ PCR array chicken innate and adaptive immune responses kit (Qiagen) was used following the manufacturer’s instructions. Each well contained specific immune related primers to detect cDNA of mRNA from genes associated with immune responses against viruses. The tested genes and the five housekeeping genes used can be found in Supplementary Table S1. Threshold cycles (Ct) calculated by the qPCRsoft software v 4.1 (Analytik-Jena AG, Jena, Germany) for each sample were loaded into a Microsoft^®^ Excel 2007 file provided by Qiagen [31]. Ct values were normalized against the housekeeping genes, and the relative expression of each gene in vaccinated and unvaccinated birds was calculated by t-test [31]. A genomic contamination control, reverse transcription controls, and positive PCR controls included in each plate for each sample were used to validate the results. Gene expression levels presenting at least a twofold difference with a significant *p*-value < 0.05 were considered relevant.

**Experimental design of experiment 2.** In the second experiment, fertile SPF (AVS Bio) and white leghorn eggs, obtained from a commercial hatchery, were hatched in the same conditions as in experiment 1. The commercial laying hens’ breeders were vaccinated repeatedly against ND and other diseases using live as well as inactivated vaccines. Maternal antibodies against NDV were detected by ELISA in commercial birds but not SPF birds [32]. After hatch, 56 birds of each type were split in 2 groups of 28 birds each, to be vaccinated when 1 day old or 14 days old. Each group of 28 birds was split into vaccinated or unvaccinated groups (*n* = 14). The experiment followed a completely randomized design in a 2 × 2 × 2 factorial arrangement, with bird type (SPF or commercial), age at vaccination (1 day or 14 days old) and vaccination status (vaccinated or control) as factors.

Vaccinated birds received the NDV LaSota strain following the same procedure as in experiment 1. Tissue sampling followed the experiment 1 protocol, and samples were collected 24 and 48 hpv and stored in DNA/RNA Shield (Zymo Research) for 24 h at 4 °C. After that, the protective reagent was discarded, and samples were stored at −80 °C.

**Sample processing and data analysis in experiment 2.** Total RNA from seven Hgs and Tcs of each group were extracted as in experiment 1, and five samples were used for all analyses based on the same quality criteria as the previous experiment. The sequencing data were uploaded to the Sequence Read Archive database (SRA accession number SAMN40632518).

Analyses of DEGs, GO, KEGG pathways and PPI were conducted as described for experiment 1 with the following modifications in the data analysis:

The DEGs analysis was performed for Hgs 24 hpv (hg24), Hgs 48 hpv (hg48), Tcs 24 hpv (tc24) and Tcs 48 hpv (tc48), comparing all vaccinated groups against all controls using the glmQLFTest for multivariate analysis using the edgeR package. Statistical analysis with edgeR requires the reference level of a factor to set a baseline for coefficient comparison. The commercial 1d control group was set as the reference level. The four lists of DEGs with an adjusted *p*-value < 0.05 for the false discovery rate (FDR) and logarithm of fold change (logFC) > ±1.5 were used to generate an upset plot with UpSetR v.1.4 [33]. This technique allowed us to visualize the number of common genes between the analyses performed. The DEGs identified in all four analyses were used to obtain the PPI and enrichment pathway map analysis, performed, as mentioned, for experiment 1. The pathways were obtained similarly to in experiment 1, and the common pathways were obtained by the same method as the one used for common DEGs. In addition, genes and pathways that were differentially expressed when considering bird types and age at infection as well as the interactions between the three main effects—vaccination, bird type and age at infection—were identified.

## 3. Results

### 3.1. Experiment 1

#### 3.1.1. Differential Expression of Genes Associated Immune Response, GO, KEGG Pathways by RNA-Seq

Due to the RNA yields and integrity achieved, not all samples of each organ, group and timepoint could be analyzed. The numbers of samples that were included are shown in Table 1. On average, each sample yielded 25 million raw read pairs with 95.5% clean reads/raw reads ratio (effective %). No DEGs were detected in the Hgs collected 12 hpv comparing vaccinated group to the control. Because of the lack of DEGs, pathways were not analyzed. At 24 hpv, 22 up- and 112 down-regulated DEGs were identified in the Hgs of vaccinated birds compared to the control group (Supplementary Table S2). The interferon-induced gene (IFI), deltex gene (DXT) (a gene that encodes for E3 ubiquitin–protein ligase present on ubiquitin–proteasome degradation pathway [34]), and tumor necrose factor receptor-associated factor (TRAF) were the innate-immune related genes up-regulated. No down-regulated genes were identified to be immune-related. The pathway enrichment analysis identified more than 50 up- and 17 down-regulated pathways. The top-fifteen significant pathways, based on their *p*-values, from vaccinated birds are shown in Supplementary Table S3. Several JAK-STAT-related pathways were up-regulated (Supplementary Table S3). The PPI did not identify protein clusters linked to immune response. For Tcs at 12 hpv, there were no up- or down-regulated DEGs on the vaccinated birds compared to the unvaccinated group.

#### 3.1.2. Differential Expression of Genes Associated with Immune Response by RT-qPCR

Samples from Hgs at 12 and 24 hpv and from Tcs at 24 hpv were compromised due to low-quality samples or not enough tissue available to perform the analysis. None of the tested genes were found to be positive in Hgs and Tcs 24 hpv by RT-qPCR. None of the genes tested in the RT-qPCR analysis were identified as being up- or down-regulated by the transcriptome analysis.

### 3.2. Experiment 2

#### Differential Expression of Genes, GO, KEGG Pathways and PPI in Response to NDV Vaccination in Harderian Glands and Tracheas

On average, each sample yielded 23 million raw read pairs with average effective % of 97.2. The number of DEGs for each main factor and interactions, categorized by organ and timepoint, is presented in Table 2. The number of DEGs from vaccination was between 294 in the Tcs at 24 hpv (168 up-regulated genes and 126 down-regulated genes) and 1175 in the Hgs at 48 hpv (114 up-regulated genes and 1061 down-regulated genes). Among the 4 vaccinated groups, there were 2169 unique DEGs. In general, vaccination resulted in an increased number of DEGs in the Hgs compared to Tcs, and more DEGs 48 hpv than 24 hpv. A similar trend was observed for age at vaccination, with more DEGs due to age in the Hgs than in the Tcs, and more at 48 hpv compared to 24 hpv. The number of DEGs due to bird type was also higher in the Hgs. However, an inverse effect was observed regarding hpv, with a higher number of DEGs due to bird type in the Hgs at 24 hpv than at 48 hpv. In the Tcs, the previously observed trend of having more DEGs at 48 hpv was seen. The number of DEGs in 14-day-old chicks compared to 1-day-old chicks and in the absence of maternal antibodies compared to the presence of maternal antibodies is shown in Supplementary Table S4.

Furthermore, there were several interactions between two main factors (vaccination and bird type/vaccination and age) and all three main effects together (vaccination with bird type, and age). For instance, when the interaction between two main effects is significant for a gene, it indicates that the expression of this gene differs depending on the type of bird and the vaccination status, e.g., a gene identified as down-regulated in vaccinated SPF birds might be up-regulated in vaccinated commercial birds or vice versa. The same applies for interactions between three main effects.

**Figure 1:**
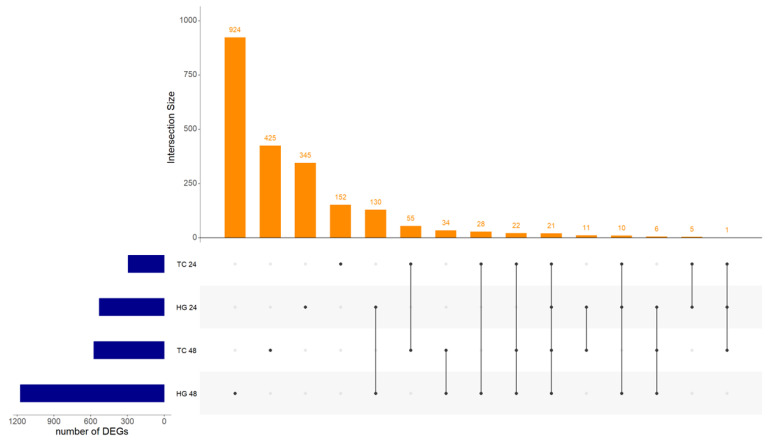
Upset plot showing intersection size between Harderian glands (Hgs) and tracheas (Tcs) at both, 24 and 48 h post-vaccination with Newcastle disease virus LaSota. Intersection size show the number of differentially expressed genes (DEGs) between the analyzed samples. Twenty-one DEGs were common between all combinations of organs and timepoints.

**Figure 2:**
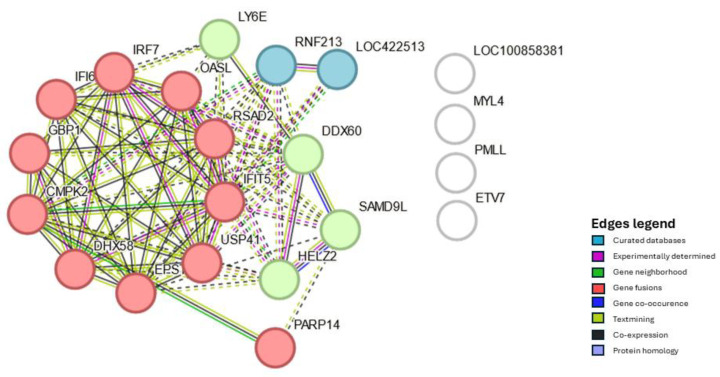
Protein–protein interaction of the core genes, i.e., genes differentially expressed in chickens in Harderian glands and tracheas 24 as well as 48 h after vaccination with Newcastle disease virus LaSota. Nodes represent proteins. Nodes with the same color are part of the same cluster. Edges represent protein–protein associations. Associations with splice isoforms or post-translational modifications are collapsed. Each node represents all the proteins produced by a single, protein-coding gene locus.

Considering all four analyses—Hgs at 24 and 48 hpv and Tcs at 24 and 48 hpv—twenty-one DEGs were present in both organs and both sampling timepoints (Figure 1). Twenty of these were consistently either up- or down-regulated in all four analyses, while one, myosin, light chain 4 (MYL4), was up-regulated at Hgs 24 hpv but down-regulated in the other three analyses. Because of this, it was used for PPI analysis but not considered a core gene. The group formed by interferon alpha inducible protein 6 (IFI6), 2′-5′ oligoadenylate synthetase-like (OASL), poly(ADP-ribose) polymerase family member 14 (PARP14), DExD/H-box helicase 60 (DDX60), DExH-box helicase 58 (DHX58), and interferon regulatory factor 7 (IRF7), among fourteen other common DEGs, will be subsequently be referred to as the core genes (Supplementary Table S5). The PPI interaction and enrichment map from the core genes formed three clusters. Those clusters were related to host innate immune response processes either by interferon, cytokine, and caspase regulation or the regulation of viral genome replication (Figure 2, Supplementary Table S6).

**Figure 3:**
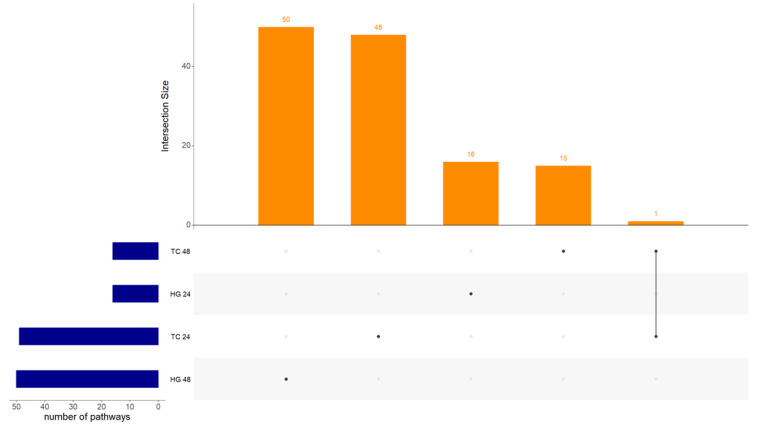
Upset plot showing intersection size between Harderian glands (Hgs) and tracheas (Tcs) at both, 24 and 48 h post-vaccination with Newcastle disease virus LaSota. Intersection size show the number of regulated pathways between the analyzed samples. No common pathways were found between all combinations of organ and timepoint.

The number of pathways for each main factor and their interactions, categorized by organ and timepoint, can be found in Table 3. There were 130 unique pathways regulated only by vaccination accounting for all organs and hpv. The regulation of these pathways did not follow the pattern observed for DEGs. Interestingly, an equal number of pathways were regulated due to age at vaccination in the Hgs compared to Tcs, and 24 hpv compared to 48 hpv. However, more pathways were regulated due to age at vaccination in the Hgs compared to Tcs, and at 48 hpv than 24 hpv. For bird type, the number of regulated pathways was higher in the Hgs than Tcs, and at 24 hpv than 48 hpv. Additionally, several pathways showed interactions between two (vaccination and bird type/vaccination and age at vaccination) or all three main effects (vaccination with bird type and age at vaccination). That means that when the interaction between two main effects is significant for a pathway, this pathway regulation differs depending on the type of bird and the vaccination status, e.g., a pathway identified as down-regulated in vaccinated SPF birds might be up-regulated in vaccinated commercial birds or vice versa. The same applies for interactions between three main effects. No pathways regulated due to vaccination were found to be common across all organs and hpv within all vaccinated groups (Figure 3).

## 4. Discussion

RNA-seq is renowned for its in-depth transcriptome analysis, whether from tissue samples or single cells. It offers a more comprehensive view of gene expression changes than PCR-based assays [35]. However, this technique is still being refined, and factors like sample size, mRNA extraction and sequencing quality, and complex statistical analysis can impact results. Conversely, RT-qPCR is often regarded as the gold standard for tracking gene transcription. However, RT-qPCR relies on well-defined target genes, so without a prior knowledge, it may fail to detect differentially expressed genes, and the selection of target genes might bias the results.

Experiment 1 aimed to compare RNA-seq and RT-qPCR results in chicks to assess the correlation between the two methods. No common DEGs were identified between the transcriptomic and RT-qPCR results. The discrepancy in the findings could be attributed to the fact that the target genes for RT-qPCR were selected before running the RNA-seq. None of the genes tested by RT-qPCR were seen to be up- or down-regulated by RNA-seq either. That means that only using RT-qPCR could lead to missing results. On the other hand, the transcriptomic approach allowed for the identification of gene expression changes without guessing what the related genes are based on prior to the analysis. Overall, RNA-seq showed potential as a helpful tool for pre-selecting genes that can be further investigated with a gold standard technique, such RT-qPCR. This could potentially lead to more consistent results.

The findings suggest that chicks vaccinated when 13 days old do not show a significant response to NDV up to 12 hpv, but the response begins to rise between 12 and 24 hpv. One of the identified up-regulated DEGs was the IFI gene, which is part of the host innate immune system and responsible for reducing viral replication by blocking single-stranded RNA virus fusion protein receptors in chicken cells [36]. This gene’s ability to inhibit viral propagation makes it a potential biomarker for immunity, particularly in relation to virus shedding. Additionally, the TRAF gene was overexpressed, indicating enhanced type I interferon production [37]. Previous research identified TRAF down-regulation caused by NDV M protein plasmids as a strategy to mock the host inflammatory response in vitro [38]. Differences between the current and cited research could be due to using different experimental models. The TRAF expression profile identified here indicates a well-functioning host early innate immune response following NDV vaccination.

Another replication strategy of viruses involves inhibiting cell apoptosis by promoting the degradation of proteins associated with cell-death up-regulation [39]. NDV specifically manipulates the ubiquitin–proteasome pathway to achieve this [40]. In the current study, there was DXT up-regulation, suggesting an increase in the degradation of proteins related to cell death by the ubiquitin–proteasome route. Even though PPI analysis did not find an increase in protein functional pathways related to these genes, gene ontology results showed an up-regulation of cell-death pathways. With that said, more studies are needed to elucidate DXT’s role in host cell death during NDV infection. All significant DEGs can be found in Supplementary Table S2.

Moving on to experiment 2, the main goal was to examine the early regulation profile of DEGs and pathways following NDV vaccination. Regarding the number of DEGs, birds vaccinated at 14 days and in the absence of maternal antibodies enhanced the number of differentially expressed genes in both organs and timepoints. The main goal of experiment 2 was to identify core genes and evaluate if they have potential as NDV vaccine biomarker candidates. Strong biomarkers are those that can be used to track protective parameters, such as innate immune response, under various conditions. In commercial poultry, there is a need for well-established NDV vaccine biomarkers that inhibit clinical signs and viral shedding. This is particularly important because it could eliminate the detection of NDV in commercial flocks, thereby reducing economic losses due to trade restrictions and depopulation. Thus, identifying innate immune genes involved in host defense or viral replication could be useful for NDV vaccine testing. Given the differences in the DEG number observed in this study, it is important to prioritize trials with commercial birds over SPF ones to identify viable vaccine biomarkers for industry application.

In that sense, experiment 2 investigated four combinations of age at vaccination and the presence of maternal antibodies as factors. The results confirmed changes in the gene expression profile between the groups, including interactions between the three main effects. The discussion will be centered in the 20 genes, the core genes, that were consistently up- or down-regulated, not only in all groups but also in the Hgs and Tcs at 24 and 48 hpv.

The transcriptomics output indicated that the core genes were involved with innate immune response regulation through changes in the interferon-I (INF-I) cascade in two ways. Firstly, by modulating genes from the pattern recognition-receptors (PRR) system, that were seen to be up-regulated. Among them, retinoic acid-inducible gene-I (RIG-I)-like receptor (RLR) and ubiquitin ligases protein families are key components of the PRR system, serving as primary tools for eukaryotic cells to recognize cytoplasmatic viruses [41]. Once a viral particle is recognized, PRR triggers several intracellular routes to activate INF-I production by regulating the gene expression of interferon-inducible genes in an attempt to reduce viral propagation [34]. In experiment 2, PRR-related genes PARP14, OASL, DHX58, and DDX60 were up-regulated together with IRF7, a non-PRR-related interferon regulator gene.

As previously mentioned, increased INF-I translation is crucial for activating the innate immune responses regulated by INF-I, including the gene expression of the IFI gene family [42]. Regarding this family, IFI6 was seen to be overexpressed in the present study. Previous research reported IFI6, also known as ISG12, to be up-regulated during NDV infection. This gene enhances mitochondrial membrane permeability and induces cell-death in an attempt to shut down several single-stranded RNA virus infections [43]. Given the importance of apoptosis in decreasing viral shedding, IFI6/ISG12 shows potential as an immunity marker for NDV vaccines, but this warrants further investigation. Other identified interferon-inducible genes, such as IFITM1 and IFIT5, are linked to NDV propagation and have potential as vaccine biomarkers. The former hinders the permeability of the host cell membrane to NDV [44], while the later directly binds to viral nucleotide sequences. The intracellular antiviral capability of IFIT5 leads to the translation of non-functional viral proteins, impacting virus replication [45].

It is worth noting that the functional profile of the core genes aligns with three clusters identified by PPI analysis. Furthermore, the enrichment map (Supplementary Table S6) has identified core genes encoding proteins that, to date, have not been specifically linked to NDV vaccinal response. Among the defense response to virus cluster genes, which include DDX60, DHX58, IFIT5, OASL, CMPK2, and RSAD2, the latter two have little characterized antiviral action upon NDV infection [10,46,47]. Moreover, USP41, which has been identified as part of the interferon regulatory factor and the negative regulation of viral genome replication clusters has not yet been linked to the immune response against NDV in chickens, according to our current knowledge.

In conclusion, using RNA-seq, this study identified key genes linked to innate immunity after vaccination against a live lentogenic NDV vaccinal strain. The PRR involving RIG-I-like receptor, their interaction with ubiquitination-linked processes, and the subsequent regulation of the immune cascade modulated by INF-I appear to be key biological processes in NDV vaccination, but they require further investigation. Furthermore, the results suggests that the core genes are connected to the host’s regulation of viral replication. This highlights them as potential marker candidates. However, the relationship between core gene expression profiles and viral shedding patterns after vaccination needs to be addressed in further experiments.

## Figures and Tables

**Table 1:** Number of samples in each treatment, per organ, and timepoint that were included in RNA-seq and RT-qPCR in experiment 1.

Organ	Hours Post-Vaccination	Control Group		Vaccinated Group	
		RNA-seq	RT-qPCR	RNA-seq	RT-qPCR
Harderian glands	12	3	0	3	2
	24	3	2	3	3
	48	2	0	3	0
Tracheas	12	3	3	2	3
	24	0	2	2	3
	48	2	0	3	0

**Table 2:** Number of differentially expressed genes (DEGs) in Harderian glands (Hgs) and tracheas (Tcs) 24 and 48 h post-vaccination (hpv) with Newcastle disease virus LaSota by main effects and their interactions.

Main Factor or Interaction	Organ and Sampling Timepoint (Hours)								Unique DEGs
	Hgs 24 hpv		Hgs 48 hpv		Tcs 24 hpv		Tcs 48 hpv		
	Up	Down	Up	Down	Up	Down	Up	Down	
Vaccination	490	39	114	1061	168	126	74	503	2169
Age	347	54	194	1069	108	201	94	547	2235
Type	1947	736	336	612	116	370	103	600	3919
Type × Age	1278	3614	412	109	324	83	521	84	5715
Vacc × Age	81	459	826	44	97	214	632	234	2333
Vacc × Type	384	1185	375	152	267	166	561	72	2692
Vacc × Type × Age	1939	403	113	188	307	312	172	464	2333

**Table 3:** Number of regulated pathways in Harderian glands and tracheas at 24 and 48 post-vaccination with Newcastle disease virus LaSota by treatment and its interactions.

Main Factor or Interaction	Organ and Sampling Timepoint (Hours)								Unique Pathways
	Hgs 24		Hgs 48		Tcs 24		Tcs 48		
	Up	Down	Up	Down	Up	Down	Up	Down	
Vaccination	16	0	0	50	30	20	8	8	130
Age	0	0	7	43	0	10	2	9	68
Type	49	1	16	34	0	10	0	5	110
Type × Age	25	25	25	25	8	8	6	6	128
Vacc × Age	0	0	50	0	2	48	15	35	149
Vacc × Type	0	50	43	7	0	0	7	0	107
Vacc × Type × Age	45	5	7	2	0	0	0	5	149

## Data Availability

The data generated in this study were submitted in the Bioproject database under ID: PRJNA1092978 and PRJNA1092705.

## Supplementary Materials

The following supporting information can be downloaded at: https://www.mdpi.com/article/10.3390/vaccines12060592/s1, Table S1: RT2 Profiler PCR Array chicken innate and adaptive immune responses kit; Table S2: Differentially expressed genes from birds vaccinated with Newcastle disease virus LaSota at 14 days old compared to the unvaccinated ones; Table S3: Pathways regulated by the differentially expressed genes from groups experimentally vaccinated with Newcastle disease virus LaSota at 14 days old compared to mock vaccinated control birds; Table S4: Effect of age and the presence of MABs on the number of DEGs; Table S5: The twenty-one common genes, core genes, from birds vaccinated with Newcastle disease virus LaSota compared to unvaccinated control. Results from the Harderian glands and Tcs at both 24 and 48 h post-vaccination; Table S6: Protein–protein interaction and its correlated enrichment pathways map based on the twenty core genes.
